# Editorial: Methods and applications in Alzheimer's disease and related dementias

**DOI:** 10.3389/fnagi.2022.1083257

**Published:** 2022-11-29

**Authors:** Alvaro Yogi, Carlos Ayala Grosso

**Affiliations:** ^1^Human Health Therapeutics Research Centre, National Research Council Canada, Ottawa, ON, Canada; ^2^Laboratory of Cellular and Molecular Pathology, Centre of Regenerative Medicine, Venezuelan Institute of Scientific Research (IVIC), Caracas, Venezuela

**Keywords:** Alzheimer's disease, dementia, methods, diagnostics, biomarker, neuroimaging

## Introduction

Alzheimer's disease (AD) is the most prevalent neurodegenerative disease worldwide. Currently, AD diagnosis is based on a multidimensional approach that involves clinical, neuropathological examination and evaluation of biomarkers. The burden of AD is further exacerbated by the fact that brain damage actually begins to develop several years before the diagnosis or even mild cognitive impairment (MCI) is observed (Long and Holtzman, 2019). Therefore, developing early and accurate diagnostic methods is urgently needed. The present Research Topic aims to highlight the latest experimental techniques and methods used to investigate fundamental questions in Alzheimer's disease and related dementias, from integrative functions to molecular and potential therapeutics.

### The challenge of the next decade: Screening of large populations with innovative approaches

The estimated total healthcare costs for the treatment of Alzheimer's disease in 2020 is estimated at $305 billion, with the cost expected to increase to more than $1 trillion as the population ages (Wong, 2020). It is proposed that early diagnosis and intervention are effective ways to reduce the burden of AD. The study by Ren et al., demonstrates the benefits of a screening program for AD in mainland China and debates the cost-effectiveness of implementing such programs for the health care system. Their report established increased health benefits and reduce the incidence of severe AD and death.

Diagnosis of AD is typically based on cognitive decline based on scores that can be affected by subjective factors. Machine learning techniques could be an attractive alternative for eliminating such biases and tracking cognitive decline and identifying risk factors in the progression of AD. Based on data from a large population-based longitudinal survey of elderly Chinese, Wang et al., were able to build a prediction model with machine learning algorithms to early identify the risk for cognitive impairment. This study demonstrates that risk-predictive models may serve in the future as a valuable tool in preventive health care.

Developments in magnetic resonance imaging (MRI), and other methods have resulted in the widespread use of brain imaging as a tool for AD diagnosis. Recently, the definition of new variables such as AD resemblance atrophy index (AD-RAI) has been introduced as a novel machine-learning-based brain atrophy biomarker for AD diagnosis (Mai et al., 2021). In contrast with single-brain regional biomarkers, the AD-RAI summarizes atrophy across multiple brain regions affected by AD. To date, the sample sizes to determine the potential clinical utility of the AD-RAI have been relatively small. Using the Minimal Interval Resonance Imaging in Alzheimer's disease (MIRIAD) database He et al., determined the same-day repeatability and 2-week reproducibility of AD-RAI and further validated its use in AD classification and prediction in a longitudinal setting.

One of the main challenges in the imaging field is regarding the reproducibility of samples acquired across multiple centers due to variety in scanner types and data acquisition protocols. A harmonization technique based on the linear mixed effect (LME) model is introduced by Kim et al., to overcome this issue. Using MRI cortical thickness measurements obtained from multiple centers, the authors showed that the score calculated by the LME method effectively compensates for the center effect across multiple datasets.

### Biomarkers and nutritional supplements

Biochemical analysis of cerebrospinal fluid (CSF) and blood are used to further support the AD diagnosis. Although levels of the core AD biomarkers are significantly lower in blood plasma when compared to CSF, the low invasiveness of its collection makes it a good tool to confirm the diagnosis of AD (Hampel et al., 2018). The study by Qin et al., expanded the number of biomarker candidates available in peripheral blood by establishing a diagnostic model based on autophagy-related genes.

Age is the strongest risk factor for AD, however, a small fraction of patients develop early-onset AD (EOAD) in their 40s or 50s (Bekris et al., 2010). Among the genes associated with EOAD, APP which is located on chromosome 21, encodes the amyloid-beta precursor protein. Its role in the pathogenesis of AD can be further highlighted by the observation that individuals with Down syndrome, caused by the presence of all or part of the third copy of chromosome 21, display large numbers of SP and NFT at an early age (Wiseman et al., 2015). Although Down syndrome is the most frequent cause of EOAD with a specific genotype there is a paucity in the characterization of MCI in these patients. This may be linked to the inherited difficulties in detecting cognitive impairment in patients with intellectual disabilities. To address this Research Topic, Fernandez et al., used the Lempel–Ziv complexity algorithm as an estimator of brain oscillatory activity and demonstrated the differences in complexity scores from Down syndrome patients with or without MCI. In addition to age, the association between diet and the risk of dementia has been extensively explored over the years (Hill et al., 2019). The beneficial effects of diet may be linked to the increased intake of micronutrients. To further study this relationship, Zhao et al. conducted a meta-analysis of 15 clinical studies to assess vitamin E intake and the risk of dementia. Interestingly, the authors indicate that although high doses of vitamin E seem to reduce the risk of dementia, side effects related to these doses should be carefully monitored.

### Amyloid precursor protein metabolism and therapeutics

Finally, hallmarks of AD include neurofibrillary tangles (NFT) and senile plaques (SP) due to the accumulation of amyloid-beta peptide (Aβ) following the sequential cleavage of amyloid precursor protein (APP) (Selkoe, 1997). As such, therapeutic approaches mediated by the inhibition of β-site APP cleaving enzyme-1 (BACE1), which is involved in the rate-limiting step of the cleavage process of APP have been proposed (Moussa-Pacha et al., 2020). Numerous BACE-1 inhibitors were tested in clinical trials, but none showed success. To overcome these failures, it has been suggested that BACE1 inhibition should be started prior to the onset of irreversible cognitive deficits. This new paradigm is tested by Blume et al., showing that treating a 4–5 months old APP knock-in mouse model with NB-360, a selective BACE1-inhibitor, halts some of the neurodegeneration phenotype present in these animals.

In summary, this Research Topic highlights novel methods and biomarkers to better diagnose and understand the pathophysiology of AD.

## Author contributions

Both authors listed have made a substantial, direct, and intellectual contribution to the work and approved it for publication.

## Conflict of interest

The authors declare that the research was conducted in the absence of any commercial or financial relationships that could be construed as a potential conflict of interest.

## Publisher's note

All claims expressed in this article are solely those of the authors and do not necessarily represent those of their affiliated organizations, or those of the publisher, the editors and the reviewers. Any product that may be evaluated in this article, or claim that may be made by its manufacturer, is not guaranteed or endorsed by the publisher.
